# Homocystinuria with Cerebral Venous Sinus Thrombosis: Excellent Recovery with Intravenous Recombinant Tissue Plasminogen Activator

**Published:** 2017

**Authors:** Vykuntaraju K Gowda, Raghunath C Nanjundappa, Hima Pendharkar, Naveen Benakappa

**Affiliations:** 1 Department of Pediatric Neurology, Indira Gandhi Institute of Child Health, Bangalore, Karnataka, India; 2 Department of Pediatric Intescivie, Sagar Hospital, Jayanagar, Bangalore, India; 3 Department of Neuroradiology, National Institute of Mental Health and Neurosciences (NIMHANS), Bangalore, India; 4 Department of Pediatrics, Indira Gandhi Institute of Child Health, Bangalore, Karnataka, India

**Keywords:** Homocystinuria, Intracranial Sinus Thrombosis, Tissue plasminogen activator

## Abstract

Hyperhomocysteinemia can cause cerebral venous thrombosis. Recombinant tissue plasminogen activator is one of the treatment options for cerebral venous thrombosis in selected cases. We present here a 7-year-old boy with homocysteinuria with stroke. MRI of brain showed cerebral venous sinus thrombosis. We successfully treated with intravenous recombinant tissue plasminogen activator. He recovered completely without any complications.

Recombinant tissue plasminogen activator can be considered one of the treatment options in cerebral venous thrombosis in homocystinura.

## Introduction

Homocystinuria is increased homocysteine in the urine (1). Increased homocysteine in the blood is a risk factor for both neurovascular and cardiovascular disorders (2).

Homocysteine level more than 50 μM is due to defects in remethylation or trans sulphuration. The estimated worldwide frequency of homocystinuria ranges from 1 case per 58 000 to 1 case per 1000000(3). Homozygous cystathionine beta synthase (CBS) deficiency presents with the major clinical manifestations of premature arteriosclerosis, thromboembolism, mental retardation, ectopialentis, and skeletal abnormalities (4).

We report a child with homocysteinuria with cerebral venous sinus thrombosis (CVST).

## Case Report

After uneventful pregnancy and normal birth, developmentally delayed seven years old boy presented with fever and altered sensorium of two days duration. He was born from consanguineously married couple. He was on spectacles for myopia since early child hood. On examination, his height was more than 97th percentile (135 cm). Supero-nasal lens subluxation with zonular dialysis was seen in both eyes. 

His Glasgow coma scale was 8/15 at the time of admission. His motor power in the lower limbs was 3/5(MRC grade). There were no signs of meningeal irritation. 

His hemoglobin was 9 gm/dl, with normal white blood cell counts and platelets. 

**Fig 1:**
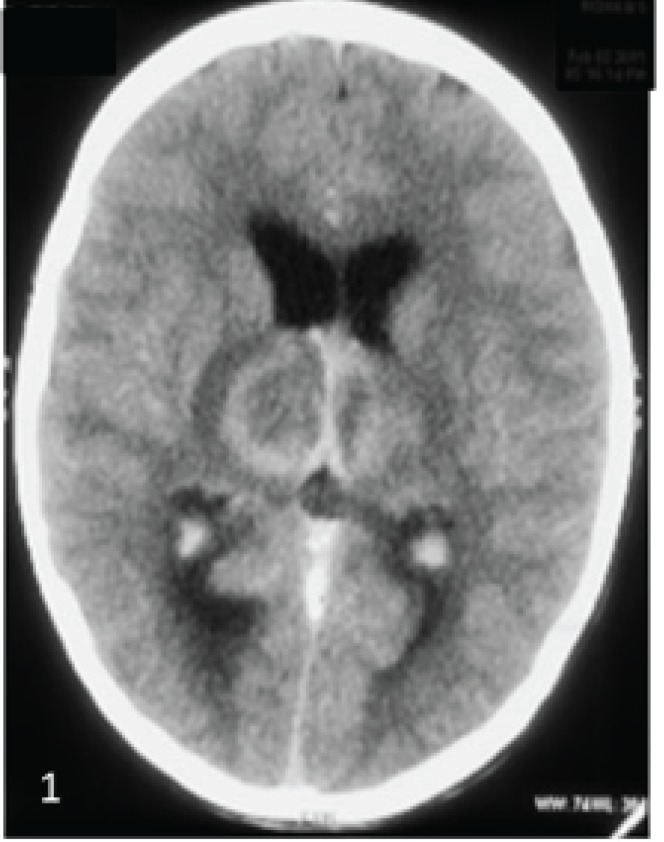
Axial non contrast CT Shows hemorrhagic infarcts with perilesional edema in bilateral thalami. Note the thrombosed hyperdense internal cerebral veins bilaterally & the thrombosed hyperdense straight sinus. Mild dilatation of lateral ventricle is noted

His serum electrolytes, liver function and renal function tests were normal. Axial non contrast CT showed hemorrhagic infarcts with peri-lesional edema in bilateral thalami (Fig 1). Mild dilatation of lateral ventricle was noted. MRI of brain shows mixed signal intensity lesions in bilateral thalami in

**Fig 2a:**
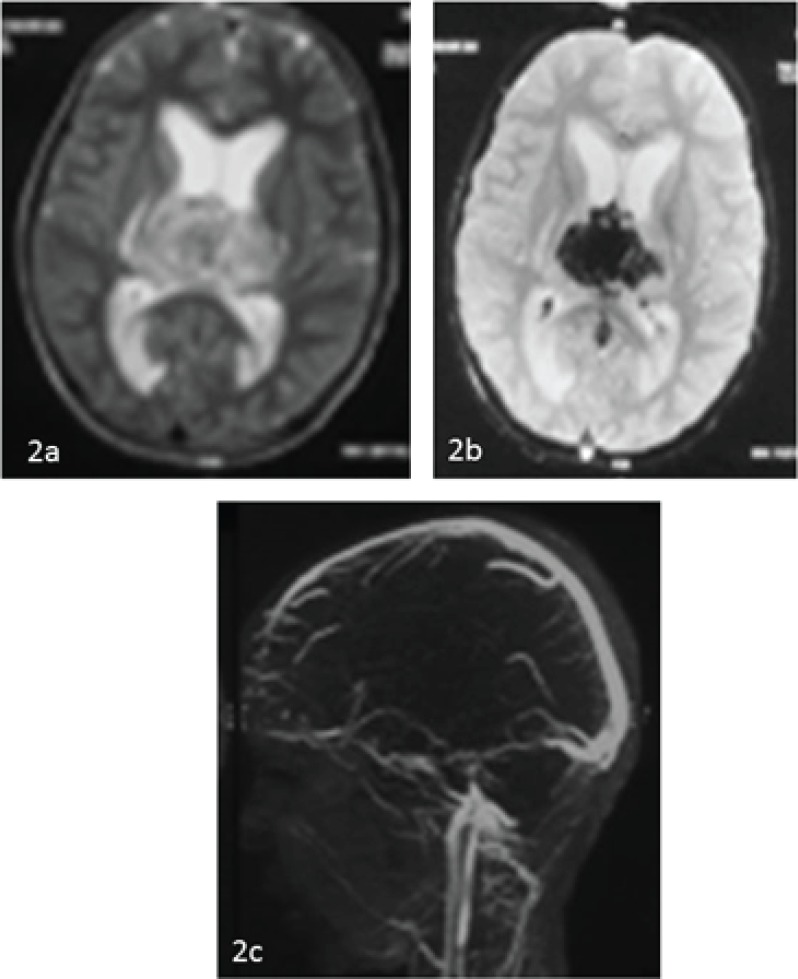
T2W axial image shows mixed signal intensity lesions in bilateral thalami.GRE axial images show the blooming of the thalamic hemorrhageSagittal view of TOF MR venogram shows non filling of the deep venous system, except for a small segment of the straight sinus

T2W axial image (Fig. 2a), blooming of the thalamic hemorrhage on GRE axial images (Fig. 2b), non filling of the deep venous system is noted except for a small segment of the straight sinus on sagittal view of TOF MR venogram (Fig. 2c). His initial Partial thromboplastin time and Prothrombin time were normal. Serum lactate, plasma ammonia, arterial blood gas was normal. His serum homocysteine (CLIA) was more than 50 μmol/l (normal- 4.9-15).

Patient was monitored in pediatric intensive care. We started on supportive measures for increased intracranial pressure and CVST was suspected secondary to homocysteinuria in view of developmental delay, lens dislocation and increased homocystine levels. Parents were counseled about the options available including heparin infusion and intravenous tissue plasminogen activator (tPA). Parents consented for intravenous tPA with intravenous heparin. Un-fractionated intravenous heparin infusion was started after bolus. Fibrinogen and partial thromboplastin time were monitored every 12 hours.

The straight sinus was catheterized with Echelon micro-catheter and 2 mg of rtPA was given as bolus,

infusion started at the rate of 2mg/h. The radiological effect of treatment was reassessed with angiogram at 12-hour intervals. rtPA infusion was discontinued after 36 hours with documentation of flow restoration in the deep venous system. Heparin anticoagulation was maintained, and converted to oral warfarin therapy after 96 hours. Child’s sensorium improved partially but hydrocephalus persisted requiring external ventricular drainage (EVD). After EVD, sensorium improved and the child recovered completely without any sequelae.

**Fig 3:**
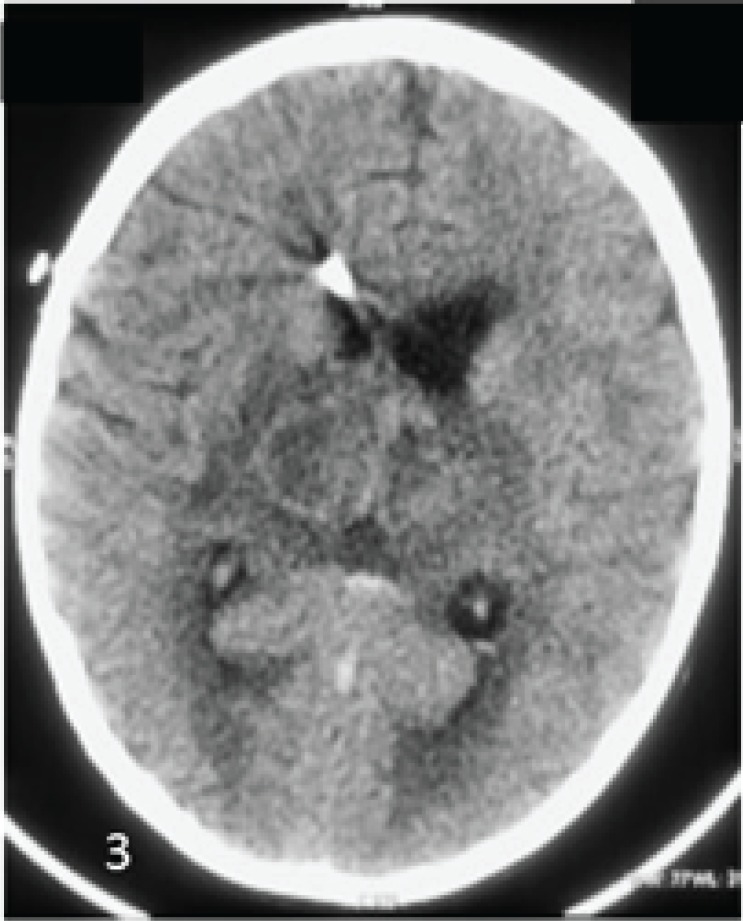
Axial non contrast CT post thrombolysis shows resolution of hyperdensity in the internal cerebral vein & the straight sinus. The thalamic hemorrhage is resolving. External ventricular drain is noted in the right frontal horn decompressing the ventricular system

Post thrombolytic CT showed resolution of hyperdensity in the internal cerebral vein & the straight sinus (Fig. 3). He also received pyridoxine, folic acid, and vitamin B12 supplementation. During his 24 months follow-up he had no recurrence of thrombosis.

## Discussion

Homocystinuria is a multisystem disorder characterized by myopia, mental retardation, downward lens dislocation, seizure, and thromboembolic events (4). 

The diagnosis of homocysteinuria is based on clinical presentation and laboratory findings. This child presented with developmental delay, lens subluxation and altered sensorium. Investigations were suggestive of CVST with increased serum homocysteine. 

There has been lack of evidence for management of CVST in children. In adults, intravenous recombinant tPA is approved therapy and it is effective in reducing disability (5) In children for acute ischemic stroke, no established treatment is available. American Heart Association Guidelines recommend tPA can be used in some children with CVST (6).

In children and adults there are no randomized control studies on thrombolysis (7), thrombectomy, or surgical decompression in CVST. These procedures have been used in isolated cases or small series in seriously ill patients (7). Tissue plasminogen activator was planned in view of low Glasgow coma scale and neuroimaging showing extensive venous thrombosis. The child received tPA for 36 hrs with un-fractionated heparin for 96 hours followed by oral warfarin. Persistent hydrocephalus required external ventricular drainage. 

The literature regarding the use of recombinant tPA in pediatric patients with acute ischemic stroke is limited to observational data. In nationwide inpatient samples, out of 2904 pediatric ischemic strokes only 1.6% of cases received intravenous and / or intra-arterial thrombolysis. 

Out of 46, 24 received only intravenous recombinant tPA (8). Similarly, registry by the International Pediatric Stroke Study (IPSS) group reported use of thrombolysis in 15(2.2%) cases among 687 ischemic strokes from 2003 to 2007(9). For 7 of the 9 cases in the IPSS registry receiving intravenous-only recombinant tPA varied substantially from the standard adult regimen of 0.9 mg/ kg at less than 4.5 hours; 3 patients were treated at 5.5, 20, and 52 hours from symptom onset and 4 received alternate dosing. Only 7 of 15 (47%) thrombolysis cases in the IPSS had favorable outcomes, with mild or no deficits at 90 days (6). 

There has been no report in children to the best of our knowledge who received tPA in CSVT due to homocysteinuria with complete recovery. An absolute indication for tPA in CVST in children is not described. 

We suggest that tPA may be considered in children with CVST with homocysteinuria.

**In conclusions, **outcome in our child with recombinant tPA in cerebral venous thrombosis with homocysteinuria are encouraging. This treatment appears to be safe without significant complications.

## Author’s Contributions

Gowda VR : Revised and approved the manuscript for important intellectual content and guarantor of the paper 

Nanjundappa RC: Diagnosis, management and writing the manuscript Pendharkar H: Designed the study, management of patient, conducted laboratory tests and analyzed the data 

Benakappa N: Supervision of the work and revision of manuscript.

All authors agreed to be accountable for all aspects of the work in ensuring that questions related to the accuracy or integrity of any part of the work are appropriately investigated and resolved.

## Conflict of interests:

None
